# Setting the Optimal Laser Power for Sustainable Powder Bed Fusion Processing of Elastomeric Polyesters: A Combined Experimental and Theoretical Study

**DOI:** 10.3390/ma15010385

**Published:** 2022-01-05

**Authors:** Ruben Vande Ryse, Mariya Edeleva, Ortwijn Van Stichel, Dagmar R. D’hooge, Frederik Pille, Rudinei Fiorio, Patrick De Baets, Ludwig Cardon

**Affiliations:** 1 Centre for Polymer and Material Technologies (CPMT), Department of Materials, Textiles and Chemical Engineering, Ghent University, Technologiepark 130, 9052 Zwijnaarde, Belgium; Ruben.VandeRyse@ugent.be (R.V.R.); cpmt@ugent.be (O.V.S.); rudinei.fiorio@UGent.be (R.F.); 2 Laboratory for Chemical Technology (LCT), Department of Materials, Textiles and Chemical Engineering, Ghent University, Technologiepark 125, 9052 Zwijnaarde, Belgium; mariya.edeleva@ugent.be (M.E.); dagmar.dhooge@ugent.be (D.R.D.); 3 Centre for Textiles Science and Engineering (CTSE), Department of Materials, Textiles and Chemical Engineering, Ghent University, Technologiepark 70A, 9052 Zwijnaarde, Belgium; 4 Department of Surgery and Anaesthesiology, Faculty of Veterinary Medicine, Ghent University, Saliburylaan 133, 9820 Merelbeke, Belgium; frederik.pille@ugent.be; 5 Soete Laboratory, Departement of Electromechanical, Systems and Metal Engineering, Ghent University, Technologiepark 46, 9052 Zwijnaarde, Belgium; Patrick.DeBaets@UGent.be

**Keywords:** laser sintering, additive manufacturing, 3D printing, energy density, degradation

## Abstract

Additive manufacturing (AM) of polymeric materials offers many benefits, from rapid prototyping to the production of end-use material parts. Powder bed fusion (PBF), more specifically selective laser sintering (SLS), is a very promising AM technology. However, up until now, most SLS research has been directed toward polyamide powders. In addition, only basic models have been put forward that are less directed to the identification of the most suited operating conditions in a sustainable production context. In the present combined experimental and theoretical study, the impacts of several SLS processing parameters (e.g., laser power, part bed temperature, and layer thickness) are investigated for a thermoplastic elastomer polyester by means of colorimetric, morphological, physical, and mechanical analysis of the printed parts. It is shown that an optimal SLS processing window exists in which the printed polyester material presents a higher density and better mechanical properties as well as a low yellowing index, specifically upon using a laser power of 17–20 W. It is further highlighted that the current models are not accurate enough at predicting the laser power at which thermal degradation occurs. Updated and more fundamental equations are therefore proposed, and guidelines are formulated to better assess the laser power for degradation and the maximal temperature achieved during sintering. This is performed by employing the reflection and absorbance of the laser light and taking into account the particle size distribution of the powder material.

## 1. Introduction

Polymeric additive manufacturing (AM) technologies are relatively recent, and their development is growing steadily. AM has mainly been used as rapid prototyping technology, but its application in manufacturing of customized end-use polymeric parts in small-series production is growing [1,2,3,4]. AM is a general term applied for describing production technologies that rely on the addition of material in a layer-by-layer fashion, which is opposed to the more commonly used subtractive production methods such as turning, milling, or drilling [5,6].

AM production methods offer remarkable advantages, such as considerably shorter times to develop prototypes, large freedom of geometrical design, and a fine resolution [6,7,8,9]. AM technologies encompass a broad range of possibilities, including fused deposition modeling (FDM) [10] or fused filament fabrication (FFF) [11], powder bed fusion (PBF) [12], pellet-extrusion-based AM [13], and stereolithography (SLA) [14]. Each of these technologies has specific advantages and has been developed for AM production of polymeric materials such as polylactic acid (PLA), acrylonitrile-butadiene-styrene (ABS), and polyamide 12 (PA12) [15].

**Figure 1:**
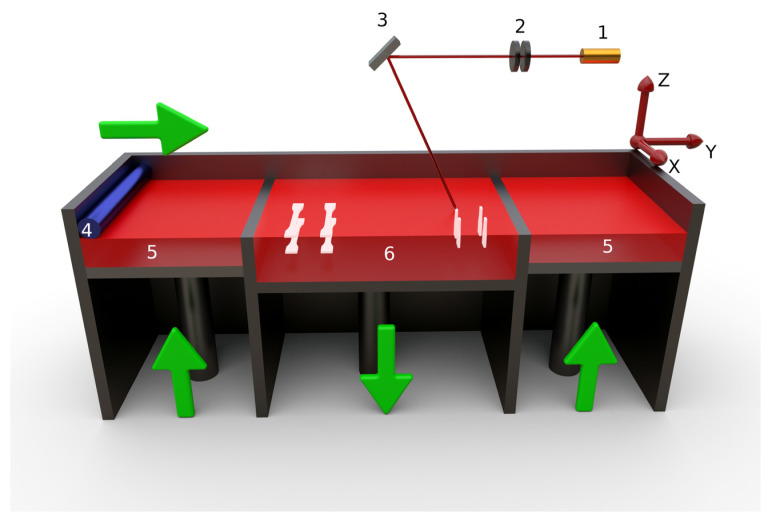
Schematic representation of the selective laser sintering (SLS) process. (1) Laser source. (2) Lenses. (3) Mirror. (4) Roller. (5) Feedstock. (6) Part bed. In the present work, printing occurs in both the XY and ZX directions.

In particular, PBF technology, of which selective laser sintering (SLS) is one of the most commonly used printing techniques, is interesting. Its working principle is illustrated in Figure 1 and consists of subsequent steps, starting with the dispersion of a powdered material over a printing bed by a roller, forming a layer with uniform thickness (*z*), followed by heating up the layer to the print bed temperature ($T_{b}$). Once (pre)heated, a laser is applied, melting specific areas of the material and forming the so-called 2D section of the desired part (since the thickness is very small). Subsequently, the print bed moves downward one layer of thickness, and a new layer of powder is supplied to the print bed. This process continues until the printing of the entire part is completed. SLS at first sight seems to be a relatively simple process, but it requires a set of processing parameters that need to be correctly set in order to successfully achieve the desired properties of the final part. These processing parameters include the print bed temperature ($T_{b}$), the removal chamber temperature ($T_{rr}$), the laser power (P), the laser speed (ν), and the laser scan spacing (S) [16].

The determination of the optimal processing parameters in SLS has proven to be a very time-consuming and costly process, as it is still predominantly based on empirical trial-and-error attempts. As a result, there is only a limited number of materials for which the optimal SLS processing parameters are known. The most commonly used materials are polyamide 11 (PA11) (e.g., Duraform EX natural, 3D-Systems), polyamide 12 (PA12; e.g., PA2200, EOS GmbH), and a few thermoplastic elastomers [17,18]. Some studies have been performed in order to develop new materials suitable for SLS technology, such as ultra-high molecular weight polyethylene (UHMWPE), polypropylene (PP), and polystyrene (PS) [12,19]. However, the demand for very specific material properties for the development and optimization of new polymeric materials limits the number of available materials for SLS. Additionally, only more recently has the focus shifted to the use of more sustainable SLS materials.

Nevertheless, significant advances have been made during the last few decades. For example, Vasquez et al. [12] proposed the stable sintering region (SSR) method for the development and optimization of new semi-crystalline polymeric materials. The SSR method consists of defining a temperature range for the SLS process based on differential scanning calorimetry (DSC) and thermogravimetric analysis (TGA). It has been suggested that this SSR encompasses a temperature range between the melt peak temperature ($T_{m}$) and the temperature of degradation at which 1% mass loss occurs ($T_{deg}$). In addition, a super-cooling region (SCR) was proposed, in which $T_{b}$ and $T_{rr}$ should be in the range between $T_{m}$ and the onset of the crystallization peak ($T_{c}{}_{onset}$). This desired range is chosen in order to easily melt the polymer with a sufficiently low laser power that keeps it in a semi-solid state after scanning of the laser has occurred [20]. The latter reduces the thermal stresses and warpage in the produced parts [21,22].

Caulfield et al. [23] investigated the effect of the energy density ($E_{D}$) on the mechanical properties of polyamide-printed parts, more specifically DuraForm^TM^ polyamide (3D Systems). They proposed an equation for the determination of $E_{D}$ ($E_{D}=P\cdot {\left(S\cdot v\right)}^{-1})$, in which P is the laser power, S is the scan spacing, and ν is the scan speed, in order to obtain adequate mechanical properties for the final printed parts. Lupone et al. [24] pushed the tuning of the processing parameters even further by optimizing the existing theoretical models. They evaluated the overall and apparent activation energy of degradation ($E_{A}$), $T_{m}$, and $T_{deg}$ and calculated the volume energy density ($E_{D}{}_{vol}$) based on the work of Starr et al. [25]. Furthermore, the specific density (ρ), packing factor (Φ), and Hausner ratio (ratio of powder tapped and bulk density) are known to influence the printability of SLS materials. Moreover, Lupone et al. [24] showed that an accurate application of the SSR method reduces the number of trial-and-error tests needed to optimize the processing parameters and leads to a better comprehension of the relation between the intrinsic properties of the materials and the optimal processing parameters.

Even though the knowledge regarding the relationship between the material properties, SLS processing parameters, and final printed part properties is gradually progressing, the development of new SLS materials still requires further investigation. An important aspect concerns sustainability both on the level of the material (e.g., being biodegradable) and on the level of the production machine being more efficient in energy use and material usage. In this sustainability context, the main purpose of the present work is to verify the effects of different SLS processing parameters on the properties of printed elastomeric polyester, a recyclable material. It is highlighted that an optimal laser power exists in terms of avoiding degradation of the processed material and that it is the combination of several SLS parameters that determines the overall macroscopic behavior.

In light of more generic process design, the suitability of improved theoretical formulas to optimize SLS parameters is also evaluated by expanding the range of the AM settings compared with the polyamide-oriented state of the art. Specific emphasis lies on the relevance of the interaction of the laser with the polymer reflectance, absorbance, transmittance, and particle size (distribution) of the powder. These characteristics have generally been overlooked in previous studies, and the results in the current work show that they should be considered in light of developing new materials and improving further modeling frameworks for SLS.

## 2. Materials and Methods

### 2.1. Materials

The polymeric material used in this research is a semi-crystalline elastomeric polyester (Amphora^TM^ 3D Polymer SP1621 Natural, Eastman Chemical Company, Kingsport, TN, USA). This material has especially been developed for powder-based AM applications. According to the technical datasheet, this polyester has a bulk density of 0.53 g·cm^−3^, a solid density of 0.99 g·cm^−3^, a Shore A hardness of 94, and in the *X*-axis of laser sintered test specimens, a tensile modulus of 143 MPa, a tensile strength of 17 MPa, and an elongation at break of 129% [26]. PA12 (PA2200, EOS) is used as a reference material.

### 2.2. Material Characterization

Thermal characterization of the polyester powder was performed using differential scanning calorimetry (DSC) and thermogravimetric analysis (TGA), conducted on a Netzsch DSC 214 and Netzsch STA 449 F3, respectively. Both analyses were performed using a heating rate of 10 °C·min^−1^ according to standards ISO 11357-1 and ISO 11358-1, respectively. A nitrogen (N_2_) atmosphere was used for all tests, with a gas flow of 40 mL·min^−1^ in the DSC and 50 mL·min^−1^ in the TGA experiments. Each tested sample had a mass of ca. 10 mg.

The specific heat capacity ($C_{p}$) of the polymer was measured according to the ASTM E1269 standard. The (overall and apparent) activation energy of degradation ($E_{A}$) of the material was obtained from TGA analysis at heating rates (β) of 10, 20, 30, and 40 °C·min^−1^ by applying the Ozawa–Flynn–Wall method [27]. This isoconversional method [27] assumes that the reaction rate at a constant conversion (α) value is only a function of the temperature [28]. From a series of decomposition curves obtained at different β values, at a given conversion, a linear correlation is observed by plotting logβ as a function of $T^{-1}$, and $E_{A}$ is obtained from the slope of the linear regression [27,29].

The particle size characteristics of the polyester powder were evaluated by optical microscopy (VHX-7000, Keyence). Microscopy images of the powders were taken, and the images were processed in ImageJ 1.52a software in order to assess the particle size and particle size distribution.

Fourier transform infrared (FTIR) spectroscopy was conducted on compression-moulded films of the polyester material. The material was dried at 80 °C for at least 8 h and subsequently compression moulded at 230 °C under ca. 3 MPa for 1 min. The FTIR spectra were obtained in a Tensor 27 spectrometer (Bruker) in transmittance mode in the interval of 4000–650 cm^−1^ using a resolution of 4 cm^−1^ and 24 scans per measurement. Two films with thicknesses of 137 and 210 µm were measured, and 5 measurements per film were collected. After the analysis, no further processing of the spectra was conducted, and the average of the 5 measurements was considered for the determination of the absorbance coefficient.

The energy absorbed by the polymer (*A*) from an incident light of a specific wavelength λ depended on the chemical structure of the material. This absorbed energy can be evaluated by the Beer–Lambert–Bouguer law:

(1) $$\tau =1-A=\frac{I}{I_{0}}=e^{-\alpha \cdot l}$$

in which τ is the transmittance (dimensionless), I is the transmitted light intensity, $I_{0}$ is the incident light intensity, l is the thickness of the sample (or pathlength), and α is the absorption coefficient. Specifically, α depends on both λ and the polymer’s chemical structure.

Colorimetric tests were conducted in an UltraScan VIS spectrophotometer (Hunterlab) with D65 Illuminant and a CIE 1964 standard calorimetric observer (10°). The results were used to calculate the yellowness index (YI) and whiteness index (WI) according to the ASTM E313 standard. The equations used are based on the CIE Tristimulus values *X*, *Y*, and *Z*:

(2) $$YI=\frac{100\cdot \left(C_{x}\cdot X-C_{Z}\cdot Z\right)}{Y}$$

(3) $$WI=Y+WI_{x}\cdot \left(x_{n}-x\right)+WI_{y}\cdot \left(y_{n}-y\right)$$

in which x and y are the chromatic coordinates of the specimen. Note that care should be taken upon interpreting x and y vs. *X* and *Y.*

The dimensionless constants in Equations (2) and (3) were selected according to the standard with regard to the D65 Illuminant and CIE 1964 colorimetric observer: $C_{x}=1.3013$, $C_{z}=1.1498$, $x_{n}=0.3138$, $y_{n}=0.3310$, $WI_{x}=800$, and $WI_{y}=1700$.

The reader is referred to Section S1 of Supporting Information for details on the mechanical testing.

The morphology of the samples obtained under different SLS conditions were analyzed by scanning electron microscopy (SEM; Phenom Pro, Thermofisher, Waltham, MA, USA). The samples were fractured after immersion in liquid nitrogen (−196 °C) for ca. 1 min, and the surfaces of the fractured samples were investigated.

### 2.3. Equations for Ideal SLS Settings: From State-of-the Art to Novel Concepts

The properties of the chosen polymer for the SLS process considerably affect the ideal window of the processing parameters. Therefore, based on the characteristics of the material and the process, one can calculate the energy for melting ($E_{m}$) and the energy for degradation ($E_{deg}$) of the polymer [24]:

(4) $$E_{m}=\left[C_{p}{}_{solid}\left(T_{m}-T_{b}\right)+\Delta H_{m}\right]\rho \cdot \Phi$$

(5) $$E_{deg}=E_{m}+\left[C_{p}{}_{melt}\left(T_{deg}-T_{m}\right)+\frac{E_{A}}{M_{m}}\right]\rho$$

in which $C_{p}{}_{solid}$ and $C_{p}{}_{melt}$ (J·(kg·°C)^−1^) are the specific heat in the solid and in the melt state, respectively, $T_{m}$ (°C) is the melting peak temperature, $\Delta H_{m}\;$(J·kg^−1^) is the enthalpy of melting, ρ (kg·m^−3^) is the specific density, Φ (-) is the packing factor of the powder (the ratio between the bulk density and the specific density), $T_{deg}$ (°C) is the degradation temperature (generally defined as the temperature at which 1% of the mass loss occurs), $E_{A}$ (J·mol^−1^) is the overall and apparent activation energy of degradation, and $M_{m}$ (kg·mol^−1^) is the mass average molar mass, which are thus all intrinsic to the polymeric material. The only factor not fully related to the polymer but to the SLS process, affecting both $E_{m}$ (J·m^−3^) and $E_{deg}$ (J·m^−3^), was the part bed temperature ($T_{b}$).

Additional formulas in the literature [24,30] aim to evaluate the effect of the laser power (P) (W), laser speed (v) (m·s^−1^), scan spacing (S) (m), layer thickness (z) (m), and laser scan count (C) (-) (i.e., the number times the laser hits the same area of the powder) on the energies correlated to the SLS process. For instance, the volume energy density ($ED_{\;vol}$) (J·m^−3^) follows from Equation (6):

(6) $$ED_{\;vol}=\frac{P}{vSz}$$

From Equations (4)–(6), the energy melt ratio (EMR) (-) and the energy melt ratio for degradation ($EMR_{D}$) (-) can be calculated by Equations (7) and (8) [30]:

(7) $$EMR=\frac{ED_{vol}}{E_{m}}$$

(8) $$EMR_{D}=\frac{E_{deg}}{E_{m}}$$

Furthermore, the laser power at which the polymer starts to degrade ($P_{D}$) can be determined by combining Equations (6) and (8) [24,30]:

(9) $$P_{D}=\frac{EMR_{D}\cdot E_{m}\cdot S\cdot \nu \cdot z}{C}=\frac{E_{deg}\cdot S\cdot \nu \cdot z}{C}$$

In order to properly evaluate $P_{D}$, one can additionally expect that the maximum temperature of the material after being exposed to the laser $T_{max}$ should not exceed $T_{deg}$ to avoid degradation. The temperatures $T_{max}$ and $T_{deg}$ are influenced by a series of factors. For example, the exposure time of the laser $t_{ex}$ (Equation (10)) directly affects $T_{max}$, with $D_{b}$ being the diameter of the laser beam (420 µm here):

(10) $$t_{ex}=\frac{D_{b}}{\nu }$$

It is further known that if the material is irradiated by a laser for a relatively short time, it shows a higher temperature near the irradiated surface than in positions far from the surface. Furthermore, the layer thickness z in the SLS process generally consists of several solid particles showing a certain packing factor. Considering that the particles on top of the layer will be directly exposed to the laser and will reach higher temperatures than the particles just below them, and that during the very short $t_{ex}$, the heat transfer between particles is negligible since the particles share a very small area of contact, $T_{max}$ can be estimated for particles with a median diameter D located on top of z.

An additional important aspect that affects the absorbed energy from the laser during the SLS process, and which is usually overlooked, is the reflectance of the incident light on the material (R). During the SLS process, the material changes from an irregular powder in the solid state to, after melting and sintering, a material with a relatively smooth surface. Consider that a slab-shaped sample R can be determined by the Fresnel expression. For the normal incidence of light (perpendicular to the surface, angle of incidence θ=0°), R is therefore given by [31]

(11) $$R=\frac{{\left(n-1\right)}^{2}}{{\left(n+1\right)}^{2}}$$

in which n is the refractive index of the irradiated material. The value of n for polymers is generally between 1.3 and 1.7 [32]. The refractive index of the polyester studied in the present work was taken to be equal to 1.52, based on the data given in various works such as Sreekanth et al. [33], Brandup et al. [32], and Jabeen et al. [34]. It is important to highlight that the value of R from Equation (11) is valid for relatively smooth materials in the SLS process (i.e., once the particles are in the molten state).

If a source of light irradiates on closely spaced particles that are large compared with the wavelength of the light, such as on SLS solid powders, the particle size and the absorption coefficient will also affect the reflectance [35,36]. Assuming that the powder particles are spherical and of similar dimensions, the hemispherical reflectance of the SLS powders ($R_{p}$) can be approximated by [36,37]

(12) $$R_{p}=\frac{1-\sqrt{1-\omega }}{1+2\cos\left(\theta \right)\sqrt{1-\omega }}$$

in which θ is the angle between the normal direction and the incident beam (for normal incidence light, cos(θ) = 1) and ω is the single-scattering albedo that follows from [35,36,37]

(13) $$\omega ≅S+\left(1-S\right)\cdot e^{-\alpha \times D}$$

in which S is the external surface scattering coefficient, or the coefficient for specular reflection from the surface of the particle averaged over all directions of incidence on one hemisphere of the external surface. Considering n values between 1.2 and 2.2, S can be approximated by [31]

(14) $$S=\frac{{\left(n-1\right)}^{2}}{{\left(n+1\right)}^{2}}+0.05$$

Furthermore, the transmittance of light through the particles $\tau_{p}$ can be calculated from

(15) $$\tau_{p}=e^{-\alpha \cdot l_{p}}$$

in which the average thickness of the particle $l_{p}$ is calculated as the thickness of a cylindrical slab with the same volume as the considered particle:

(16) $$l_{p}=\frac{\frac{4}{3}\pi {\left(0.5\cdot D\right)}^{3}}{\pi {\left(0.5\cdot D\right)}^{2}}$$

Taking into account the previously introduced R, $R_{p}$, and $\tau_{p}$ relations, novel SLS design equations are within reach. In the present work, we explore such equation development considering, for simplicity, a single particle with a specific size so that there is no formal need for a packing factor. As explained in Supporting Information, from the volume of a powder particle $V_{p}$, the projected area of the laser beam $Ar_{b}$, and the projected area of the powder particle $Ar_{p}$, one can calculate the laser power for degradation $P_{D_{ref+abs}}$, and the maximal temperature reached in the particle $T_{max}$:

(17) $$P_{D_{ref+abs}}=\frac{V_{p}\cdot \rho \cdot Ar_{b}\cdot \left[C_{p}{}_{solid}\cdot \left(T_{m}-T_{b}\right)+\Delta H_{m}\right]}{t_{ex}\cdot \left(1-R_{p}\right)\cdot \left(1-\tau_{p}\right)\cdot Ar_{p}}+\frac{V_{p}\cdot \rho \cdot Ar_{b}\cdot C_{p_{melt}}\cdot \left(T_{deg}-T_{m}\right)}{t_{ex}\cdot \left(1-R\right)\cdot \left(1-\tau_{p}\right)\cdot Ar_{p}}$$

(18) $$T_{max}=T_{m}+\left[\frac{P\cdot \left(1-R\right)\cdot \left(1-\tau_{p}\right)\cdot Ar_{p}}{Ar_{b}\cdot \rho \cdot V_{p}\cdot C_{p_{melt}}}\right]\cdot \left\{t_{ex}-\frac{\left(V_{p}\cdot \rho \cdot Ar_{b}\right)\cdot \left[C_{p}{}_{solid}\cdot \left(T_{m}-T_{b}\right)+\Delta H_{m}\right]}{P\cdot \left(1-R_{p}\right)\cdot \left(1-\tau_{p}\right)\cdot Ar_{p}}\right\}$$

In Equation (17), the subscripts “*ref*” and “*abs*” are explicitly used in order to highlight the consideration of reflectance and absorbance compared with Equation (9).

### 2.4. Experimental SLS Printing Specifications

The AM experiments were performed with an EOS Formiga P10 machine (EOS GmbH Electro Optical Systems, Krailling, Germany) using a CO_2_ laser with a wavelength in the range of 10.2–10.8 µm and a diameter of the focused beam of approximately 0.42 mm. In order to evaluate the influence of the processing parameters, three types of setting conditions were initially selected, as shown in Table 1. The laser power (P) was varied between 13 and 28 W. Parts printed with the same settings were distributed equally along the print bed, taking into account that the parts were far enough from each other and far enough from the side of the print bed in order to avoid boundary effects. The scan count was maintained at 1, as this is the standard setting of the SLS machine.

Parts with dimensions of ca. 10 × 10 × 5 mm were printed according to the settings mentioned in Table 1. These parts were subsequently characterized by their density and colorimetric properties. Afterward, tensile, flexural, and impact test specimens were made based on the third type of setting conditions in Table 1 but only at a laser power P = 21 W and using different layer thicknesses (z equal to 0.10 or 0.18 mm), and build orientations (XY- or ZX-plane, as seen in Figure 1).

## 3. Results and Discussion

Emphasis is first put on the polyester material properties and subsequently on the SLS process, considering three types of production settings and inspecting the macroscopic properties of the printed parts. Finally, the experimental results are utilized to improve the predictive nature of the theoretical equations introduced above, and guidelines are formulated for future developments.

### 3.1. Analysis of Polyester Material Properties

As shown in Table 2 (polyester column), the DSC results for the polyester material showed a melting peak temperature (*T_m_*) of 210.8 °C and a melting enthalpy ($\Delta H_{m}$) of 20.18 J·g^−1^. The specific heat capacities were $C_{p_{solid}}$ = 2376 J·(kg·°C)^−1^ and $C_{p_{melt}}$ = 2524 J·(kg·°C)^−1^. $M_{m}$ was assumed to be 31129 g·mol^−1^ [38]. The TGA results for the polyester material reveal that the temperature at 1 % of mass loss ($T_{deg}$) was 348 °C. The overall and apparent activation energy of degradation ($E_{A}$) was calculated as described in Section 2.2. Considering a conversion of 1% (α=0.01), the activation energy of degradation equaled $E_{A}=143.0\;\mathrm{kJ}\cdot {\mathrm{mol}}^{-1}$.

It can be further seen in Table 2 that $T_{m}$ and $T_{c}$ were slightly higher for the polyester compared with PA12 (last column), while PA12 showed a much higher $\Delta H_{m}$ and $E_{A}$ as well as higher $C_{p_{solid}}$ and $C_{p_{melt}}$ values.

**Figure 2:**
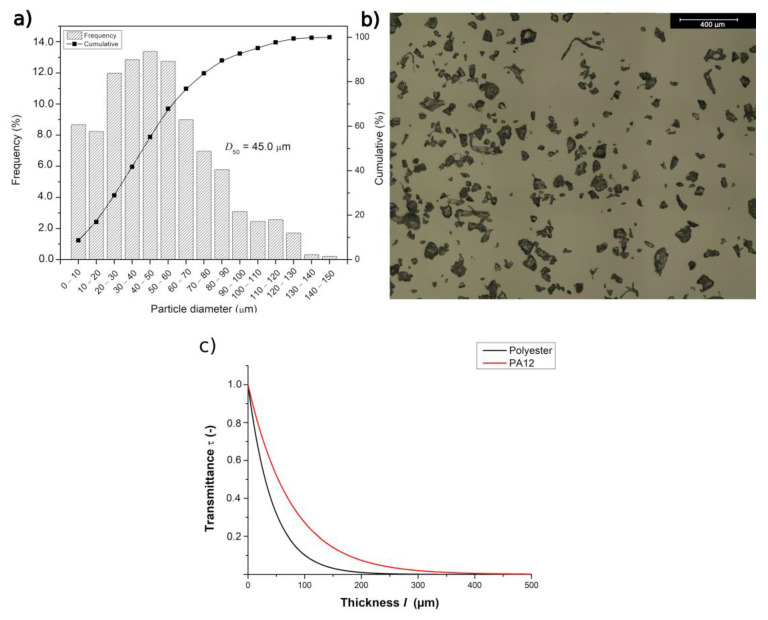
(**a**) Particle size distribution for the polyester Amphora material and (**b**) optical microscopy image showing general powder geometries. (**c**) Transmittance τ as a function of the thickness l for the polyester and PA12. Calculated results are based on Equation (15).

Figure 2b shows one of the six analyzed pictures for the particle size distribution measurement. It follows that the median particle diameter ($D_{50}$) of the polyester was 45 µm, with a maximum particle diameter of ca. 150 µm, as is also clear from Figure 2a. Furthermore, the particles showed a broad size distribution, as can also be seen in Figure 2a. The particles presented sharp corners, as shown in Figure 2b, indicating that the powder was likely produced by cryogenic milling [18,40,41,42].

The Fourier transform infrared spectra (FTIR) of compression-moulded polyester films investigated in this study indicated a transmittance (τ) of 4.61 ± 0.1% for a film of thickness = 137 ± 5 μm, and τ = 0.77 ± 0.1% for a film of thickness equal to 210 ± 5 μm at λ = 10.6 μm, being the average wavelength of the laser used for sintering and corresponding to a wavenumber of ca. 943.2 cm^−1^. The absorption coefficient α for the polyester was 22,816 ± 1756 m^−1^. PA12 is the most extensively investigated polymeric material used in SLS today. For PA12, at λ = 10.6 μm, the α value was found to be 13,000 m^−1^ [43]. For comparison reasons, Figure 2c shows the calculated transmittance of polyester and PA12 as a function of the sample thickness. One can clearly notice that there was an exponential decrease of the transmittance with an increasing sample thickness and that, when considering the same value of l for a large range of thicknesses, the polyester showed substantially lower transmittance values compared with PA12. A lower value of τ at an identical value of l implies that the polyester absorbed more laser energy than PA12. Therefore, the polyester heated up at a faster rate and could start degrading at lower values of P compared with PA12.

### 3.2. Effect of SLS Settings on the Printed Material Properties

Emphasis was first put on the typical settings for AM of PA12 (PA2200, EOS), with the first setting shown in more detail in Table 1. This was performed since some characteristics of this polymer and the selected polyester were rather similar (e.g., ρ, Φ, and $T_{deg}$) (see Table 2). The parts printed using the first setting presented important deformations and thus deviations from the expected plate shape (ca. 10 × 10 × 5 mm). These deformations could be attributed to the low removal chamber temperature ($T_{rr}$) compared with the crystallization temperature of the polyester ($T_{c}$). If $T_{rr}$ was below $T_{c}$, the part shrinkage was uncontrolled and occurred in a faster way [44]. Furthermore, although the median particle diameter ($D_{50}$) of the polyester powder was similar to that of PA12, the particle size distribution measurement of the polyester indicated the presence of particles larger than 100 μm, as shown in Figure 2a. Hence, the layer thickness z=0.10 mm could be considered too small to allow for an adequate process without deformation of the printed parts during the dispersion of a new layer of powder over the printing bed (cf. Figure 1). Hence, the layer thickness for the second and third setting conditions in Table 1 was increased compared with the first setting, although one should realize that a complete set of parameters defines the overall SLS performance. Due to the differences in $T_{m}$ and $T_{c}$, both $T_{b}$ and $T_{rr}$ were increased for the second and third settings. In total, several variations were included in the present work to enable better verification of the generic nature of the current theoretical models. Despite how $T_{rr}$ and $T_{b}$ could be set to even higher values according to the SSR and SCR methods [21,30], the maximum values for $T_{rr}$ and $T_{b}$ were practically 155 °C and 172 °C due to the heat fuses used in the machine. Increasing $T_{b}$ and $T_{rr}$ should lead to an increase in the melt volume rate and decrease the (thermoplastic) viscosity, reducing the warpage and thermal stresses in the final parts [12]. For the thirrd setting conditions, test specimens were also printed with part orientations in both the XY- and ZX-planes to better understand the inter- and intralayer adhesion (cf. Figure 1). This was performed with a layer thickness (z) of 0.10 and 0.18 mm and a laser power P = 21 W. Note that the laser power of 21 W was still far below the calculated $P_{D}$ (see in detail in the next subsection), and therefore the polyester powder should (at least theoretically) not have degraded. The flexural and impact test specimens obtained using z = 0.10 mm for the ZX-plane presented severe deformations upon comparison with the expected final dimensions and shape. Here, the reliable mechanical characterization of these specimens was impossible, highlighting the future need of an interlayer SLS design. The results of the mechanical properties that could be recorded are summarized in Table 3.

It is shown in Table 3 that both z and the build orientation (Figure 1) affected the mechanical properties. The test specimens built in the XY direction showed higher tensile strengths and tensile strains at break. Increasing z led to a decrease in the tensile properties, consistent with the recent FFF work of Fernandez et al. [45]. The flexural modulus showed a small dependency on both z and the build orientation, while the impact strength showed a slight increase if z was increased in the XY-plane. The decrease in both the tensile and flexural moduli can be explained by poorer interlayer bonding upon using a higher layer thickness, thus overruling the aforementioned particle size effect. These results are also similar to the results found by Caulfield et al. [23] and can be explained by a decreased volume energy density ($ED_{vol}$) with the increase in layer thickness. One could thus expect that a higher laser energy should therefore be used upon using a higher layer thickness to keep $ED_{vol}$ constant, as expressed in Equation (6).

Diverging from the results of Caulfield et al. [23], the impact strength of the printed parts tended to increase with the decrease in $ED_{vol}$, and the strain at break was higher for the XY-oriented parts compared with the ZX-oriented parts. The higher impact strength observed for the samples obtained at z=0.18 mm and the XY-orientation was likely related to the larger number of voids, and thus a lower density was found in the samples produced using a higher z, which facilitated deformation and energy dissipation, again reminiscent of the interlayer aspect. The higher strain at break as well the higher tensile strength were noted by Hofland et al. and could be attributed to anisotropy. This anisotropy was caused by the layer-wise production and how the stresses and forces were oriented according to the interlayer bonding [46]. In a similar fashion, Caulfield et al. found a lower impact strength for parts printed according to the ZX-plane [23]. The tensile and density results also showed the same tendencies as those found by Rüsenberg et al. [47]. However, these authors did not observe substantial differences in the impact strength for samples obtained at different layer thicknesses, while the impact strength decreased for the XZ-oriented parts. This decrease in impact strength was most likely due to the orientation of the applied force compared with the orientation of the interlayer bonding within the manufactured parts.

It is important to highlight that during SLS of the parts in the present study, independent of all other setting conditions, fumes were observed upon using a laser power (P) equal to or above 21 W, suggesting that significant degradation phenomena then occurred. Similar to this observation, Dadbakhsh et al. [48] observed fumes upon sintering a polyurethane (TPU) powder in which, due to polymer degradation, the release of fumes increased in cases of high ED values.

**Figure 3:**
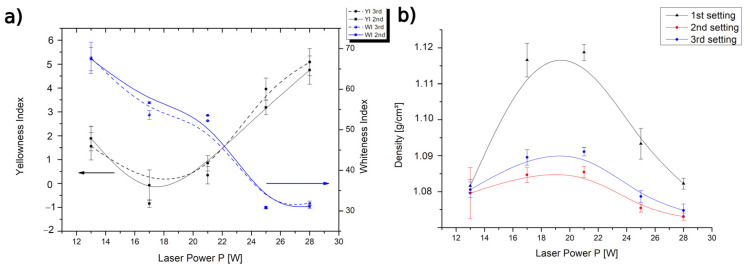
(**a**) Yellowness index (YI) and whiteness index (WI) for the second and third setting conditions at different laser powers (*P*) (Table 1). (**b**) Density of the samples obtained using the three SLS settings at different laser powers (P) (Table 1).

The colorimetric results are shown in Figure 3a. A substantial decrease in the whiteness index of the sintered parts can be noticed upon increasing the laser power, indicating that chemical modifications in the polymer structure were occurring. The YI showed an initial drop when P increased from 13 to 17 W, followed by a gradual increase with the increasing P. The initial decrease in the YI was most likely related to a lower surface roughness of the sample obtained at P = 17 W compared with the one made at 13 W, since a lower sintering efficiency was expected when using the lowest laser power. It has been previously observed that decreasing the surface roughness increases the luminance (luminance is represented by “Y” in Equation (2)) [49]. Therefore, an increase in Y leads to a reduction in the YI. However, if P > 17 W, a gradual increase in the YI of the produced parts was observed upon increasing P, indicating that a modification in the color characteristics (“X” and “Z” in Equation (2)) of the material was occurring due to degradation. The WI and YI results agreed with the conclusions drawn by Dadbakhsh et al. [48].

From Figure 3b, one can observe that the density values were higher if z = 0.10 mm (first setting) in comparison with the results found for z = 0.18 mm (second and third setting). This means that as long as the larger particles did not induce any deformations to the final part, one could still consider lower *z* values. Furthermore, the increase in P up to 21 W led to an increase in density, and with P > 21 W, the density results tended to decrease again due to material degradation. The decrease in density with an increase in P was also found by Ho et al. [50] at too high an energy input for the sintering of polycarbonate. Furthermore, a higher part bed temperature ($T_{b}$) also increased the density, as was clear when comparing the results of the third setting ($T_{b}$ = 172 °C) with those of the second setting ($T_{b}$ = 169 °C) (Table 1). This finding is in accordance with those of Tontowi et al. [51]. Furthermore, for all settings, the maximum density arose for a similar *P* (close to 21 W in Figure 3b), but this specific *P* was not exactly the same as the minimum value of the color change (Figure 3a).

**Figure 4:**
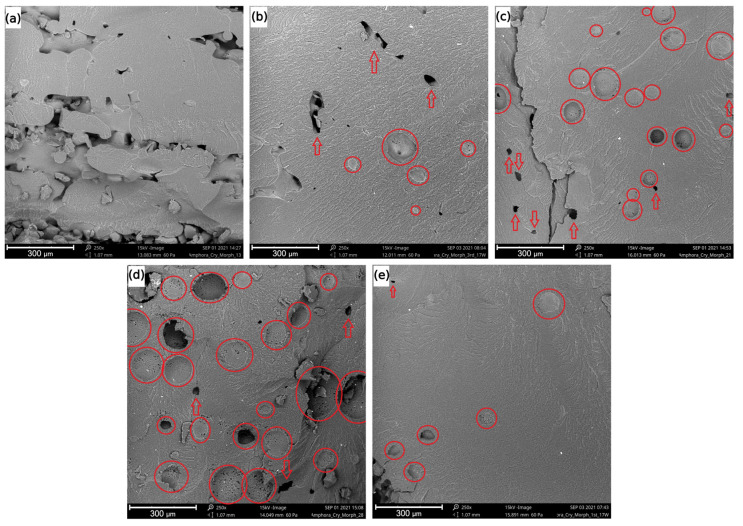
SEM images of the samples obtained under different conditions. Third setting condition: (**a**) P = 13 W; (**b**) P = 17 W; (**c**) P = 21 W; (**d**) P = 28W. First setting condition: (**e**) P = 17 W. Circles indicate voids formed due to degradation, and arrows indicate voids formed due to incomplete sintering.

With SEM analysis, the morphology of the samples obtained at different laser powers *P* (13, 17, 21, and 28 W) was investigated. The results are presented in Figure 4. This figure clearly indicates that the laser power affected the morphology of the samples. The sample obtained at *P* = 13 W (Figure 4a) showed incomplete melting of the powder and several irregular voids formed due to incomplete sintering. The sample obtained at *P* = 17 W (Figure 4b) displayed a more uniform fracture surface, indicating an improved sintering process. However, spherical voids were observed, indicating degradation of the polymer.

The samples obtained using *P* = 21 W and 28 W (Figure 4c,d, respectively) showed fracture surfaces similar to the one obtained at 17 W, albeit with a larger number of voids that were also larger in diameter, indicating a more severe degradation with the increase in *P*. Figure 4e shows the morphology of the sample from the first setting condition at *P* = 17 W. One can observe the presence of spherical voids caused by degradation of the material, similar to Figure 4b (17 W, third setting condition), but almost no voids due to inefficient sintering were observed. All these results thus corroborated the density results.

### 3.3. Theoretical Interpretation and Further Optimization

The results of the theoretical calculations for the energies and laser power for degradation ($P_{D}$) are presented in Table 4. According to the results shown in Table 4, the polyester should have started degrading at P ≥ 25.7 W during the first setting. Furthermore, the polymer should not degrade under any value of P for the second or third setting conditions, since all $P_{D}$ values were higher than the maximum laser power in this study. The aforementioned experimental colorimetric, density, and morphological results indicate, however, that the polymer did indeed partially degrade at P ≥ 17 W. This implies that the theoretical formulas currently in use fail at predicting $P_{D}$. They are much more conservative than reality, as they predict that the material should degrade once P ≥ 25.7 W for the first setting or once P ≥ 37.0 W or 36.6 W for the second and third settings, respectively. The discrepancy between the theoretical and experimental results partially explains why the development of new SLS materials is still is a long, time-consuming, trial-and-error process.

By examining Equation (5) for the energy for degradation ($E_{deg}$), one can observe that the packing factor (Φ) was not considered, while Φ was taken into account for the evaluation of $E_{m}$ (Equation (4)). As a result, Equation (5) neglected the presence of voids in the powder, leading to higher values of $E_{deg}$. In the current study, a modified (“mod”) calculation for $E_{deg}$ is therefore proposed:

(19) $$E_{{deg}_{mod}}=E_{m}+\left[C_{p_{melt}}\left(T_{deg}-T_{m}\right)+\frac{E_{A}}{M_{m}}\right]\rho \times \Phi$$

With Equation (19), the values of the energy for degradation ($E_{deg}$), energy melt ratio for degradation ($EMR_{D}$), and laser power for degradation ($P_{D}$) for the polyester material were recalculated (see Table 5).

When comparing the data from Table 4 with those in Table 5, one can observe that the application of Equation (19) resulted in lower values for $E_{deg}$, $EMR_{D}$, and $P_{D}$, as expected. The new values of $P_{D}$ found for the second and third setting conditions partially supported the colorimetric and density results previously discussed, as the application of P > 17 W led to substantial degradation. However, for the first setting, the value of $P_{D}$ was rather low (only 15.6 W), indicating that a considerable degradation should have occurred at P = 17 W, which was not observed. Therefore, Equation (19) and the other equations used for the evaluation of $P_{D}$ still should be considered approximations.

A factor that has been neglected for the tuning of process parameters for SLS is the interaction of the laser, presenting a particular wavelength (λ), with the polymer, as reflection, absorption, and transmittance of the light will occur. The existing theoretical models do not consider the absorptivity coefficient of the laser for the polymer of interest (cf. Figure 2c). This partially justifies the failure of the theoretical models upon predicting the optimal SLS conditions for materials other than PA12. Another factor was the packing factor, which was considered a fixed factor and taken from the datasheet (0.535).

**Figure 5:**
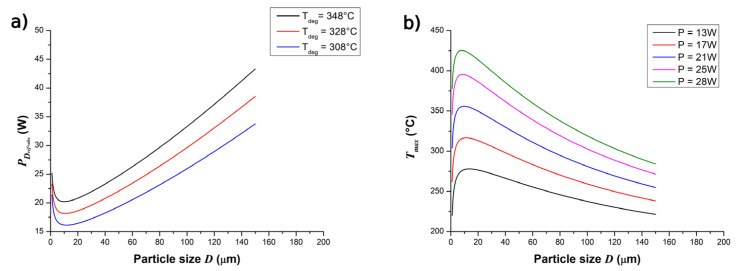
(**a**) Laser power for degradation $P_{D_{ref+abs}}$ as a function of the individual particle size D, considering different degradation temperatures ($T_{deg}$ = 348, 328, and 308 °C). (**b**) Maximal temperature $T_{max}$ as a function of particle size D, upon considering different laser powers for sintering (*P* = 13, 17, 21, 25, 28). At *T_b_ =* 172 °C.

Therefore, it is worthwhile to explore the relevance of the novel equations for $P_{D_{ref+abs}}$ and $T_{max}$ (Equations (17) and (18)). The effect of the particle size, according to the proposed equations, is shown in Figure 5. The range of D corresponded to the particle size distribution presented in Figure 2a. As shown in Figure 5, there was a specific particle size (ca. 10 μm) for which the laser power $P_{D_{ref+abs}}$ reached a minimal value (Figure 5a), leading to a maximal temperature $T_{max}$ in the polymeric material (Figure 5b). From the results shown in Figure 5, one can, for example, observe that with $T_{deg}$ = 348 °C (temperature at which 1% mass loss is observed) and D ≅ 10 μm, the calculated laser power for degradation $P_{D_{ref+abs}}$ was approximately 20 W. This value was still above the value of P = 17 W, in which degradation was observed in the morphological results (see Figure 4). However, one could expect that the thermal degradation of the polymer, with the generation of decomposition gases, started at a substantially lower temperature than 348 °C.

To illustrate the effect of $T_{deg}$ on $P_{D_{ref+abs}}$, Figure 5a also presents the theoretical values of $P_{D_{ref+abs}}$ with respect to D at three $T_{deg}$ values (348, 328, and 308 °C). One can notice that the minimum values of $P_{D_{ref+abs}}$ at D ≅ 10 μm reduced to ca. 18 W and 16 W at $T_{deg}$ = 328 °C and 308 °C, respectively. Therefore, $T_{deg}$ should be defined in a lower temperature range than the one at which 1% of mass loss is observed. Further insights were obtained from Figure 5b, in which the increase in P led to an increase in $T_{max}$. The peaks of $T_{max}$ found at different P values occurred in the range of 7.9 ≤ D ≤ 13.4 μm. Figure 5b indicates that $T_{max}$ ≅ 317° C at P = 17 W and D ≅ 10 μm. Since only a slight degradation was observed at P = 17 W independent of the value of *z*, this indicates that thermal decomposition of the polyester occurred at temperatures of ca. 317 °C, the maximum $T_{max}$ found at this laser power. This result also supports that the value of $T_{deg}$ should be defined as lower than the temperature at which 1% of mass loss occurs

Finally, when considering the proposed models for estimation of the SLS parameters, the energy necessary for printing could be reduced by properly selecting the particle size range that more efficiently absorbed the laser power. These models also indicate that a narrower particle size distribution around an adequate median would be beneficial for selecting the optimal sinter parameters for a given material, because the variation in $T_{deg}$ and $P_{D_{ref+abs}}$ would then be smaller.

## 4. Conclusions

The influence of the SLS processing parameters on the colorimetric, morphological, physical, and mechanical properties of AM parts made with a thermoplastic elastomer polyester were evaluated. A higher part bed temperature $T_{b}$, a lower layer thickness z, and the XY-plane build orientation resulted in a higher density and better mechanical properties. However, the optimal value of z depended on the particle size distribution, with particles that were too large inducing unacceptable deformations during printing.

Evaluation of the colorimetric characteristics, specifically the yellowness and whiteness indices, is an effective approach to assess the degradation of the material in the manufactured parts. Degradation of the material can also be evaluated by the evaluation of the density and by microscopic analysis. In any case, a most suitable laser power value can be put forward, which in the current work was around 17–20 W.

In general, the predicted laser energy inducing degradation ($P_{D}$), even the optimized one in the present work, was still approximate, so further optimization is still recommended.

The defined temperature of degradation $T_{deg}$ should be lower than the currently recommended temperature at which 1% mass loss occurs, as it was noticed from SEM microscopy that degradation started well before the temperature at which 1% mass loss occurs.

The novel modeling concept revealed that a *D* range existed in which $P_{D}$ was at its minimum and $T_{max}$ reached its maximum, indicative of design possibilities for the SLS process. Selecting the range of D in which $P_{D}$ is at its minimum could also reduce the energy necessary for printing, reducing costs and increasing the sustainability label of SLS.

## Figures and Tables

**Table 1:** Initial experimental SLS parameter settings. Bold entries are variables vs. first setting.

Parameter	1st Setting	2nd Setting	3rd Setting
Part bed temperature ($T_{b}$) (°C)	169.0	169.0	**172.0**
Removal chamber temperature ($T_{rr}$) (°C)	150.0	**155.0**	**155.0**
Laser power (P) (W)	13, 17, 21, 25, 28	13, 17, 21, 25, 28	13, 17, 21, 25, 28
Laser speed (v) (mm·s^−1^)	2500	2500	2500
Scan count (C) (-)	1	1	1
Scan spacing (S) (mm)	0.25	**0.20**	**0.20**
Layer thickness (z) (mm)	0.10	**0.18**	**0.18**

**Table 2:** Properties of the polyester studied in the present work and, for comparison, of PA12 [30].

Property	Polyester	PA12 [30]
$\mathrm{Melting}\;\mathrm{peak}\;\mathrm{temperature}\;(T_{m})$ (°C)	210.8 ^3^	185.0
$\mathrm{Crystallization}\;\mathrm{peak}\;\mathrm{temperature}\;(T_{c})$ (°C)	164.1 ^3^	149.1 ^3^
$\mathrm{Specific}\;\mathrm{heat}\;\mathrm{capacity}\;\mathrm{of}\;\mathrm{the}\;\mathrm{solid}\;(C_{p_{solid}})$ (J·(kg·°C)^−1^)	2376 ^3^	3155
$\mathrm{Specific}\;\mathrm{heat}\;\mathrm{capacity}\;\mathrm{of}\;\mathrm{the}\;\mathrm{melt}\;(C_{p_{melt}})$ (J·(kg·°C)^−1^)	2524 ^3^	3250
$\mathrm{Enthalpy}\;\mathrm{of}\;\mathrm{melting}\;(\Delta H_{m})$ (J·kg^−1^)	20,180 ^3^	97,200
Bulk density (Q) (g·cm^−3^)	0.530 ^1^	0.485
Specific density (ρ) (g·cm^−3^)	0.99 ^1^	0.97
Packing fraction (Φ) (-)	0.535 ^1^	0.500
$\mathrm{Temperature}\;\mathrm{of}\;\mathrm{degradation}\;(T_{deg})$ (°C)	348 ^3^	325
$\mathrm{Activation}\;\mathrm{energy}\;\mathrm{of}\;\mathrm{degradation}\;(E_{A})$ (kJ·mol^−1^)	143.0 ^3^	279.5
$\mathrm{Mass}\;\mathrm{average}\;\mathrm{molar}\;\mathrm{mass}\;(M_{m})$ (g·mol^−1^)	31,129 ^2^	18,800
$\mathrm{Median}\;\mathrm{particle}\;\mathrm{diameter}\;(D_{50})$ (µm)	45 ^3^	56

^1^ From [39]. ^2^ From [38]. ^3^ Data evaluated by the authors.

**Table 3:** Mechanical properties of polyester parts printed according to the third setting conditions from Table 1, with orientations defined in Figure 1. The general trend in tensile properties can also be seen from the stress–strain diagram shown in Supporting Information, Section S3.

Property	z = 0.10 mm		z = 0.18 mm	
	XY	ZX	XY	ZX
Tensile strength (MPa)	14.3 ± 0.1	9.7 ± 0.2	11.3 ± 0.4	5.9 ± 0.9
Tensile modulus (MPa)	133.9 ± 2.5	131.0 ± 2.0	96.7 ± 7.7	105.6 ± 3.6
Tensile strain at break (%)	60.5 ± 4.0	16.3 ± 1.0	36.1 ± 3.0	7.5 ± 2.2
Flexural modulus (MPa)	91.4 ± 2.6	*	82.6 ± 3.5	87.5 ± 3.6
Charpy impact strength (kJ·m^−2^)	8.18 ± 1.12	*	10.45 ± 0.94	4.38 ± 0.47

* Analyses not performed due to deformations of the test specimens during the SLS process.

**Table 4:** Calculated results for the energies ($E_{m}$, $E_{deg},$ $EMR_{D}$, $ED_{\;vol}$, and EMR) and laser power for degradation ($P_{D}$) related to the SLS process of the polyester (Equations (4)–(8)).

Theoretical Property	First Setting					Second Setting					Third Setting				
$\mathrm{Energy}\;\mathrm{for}\;\mathrm{Melting}\;(E_{m})$ (mJ·mm^−3^)	63.3					63.3					59.5				
$\mathrm{Energy}\;\mathrm{for}\;\mathrm{Degradation}\;(E_{deg})$ (mJ·mm^−3^)	410.7					410.7					406.9				
$\mathrm{Energy}\;\mathrm{Melt}\;\mathrm{Ratio}\;\mathrm{for}\;\mathrm{Degradation}\;(EMR_{D})$ (-)	6.485					6.485					6.833				
$\mathrm{Laser}\;\mathrm{Power}\;\mathrm{for}\;\mathrm{Degradation}\;(P_{D})$ (W)	25.7					37.0					36.6				
	Laser power (P) **(W)**														
	**13**	**17**	**21**	**24**	**28**	**13**	**17**	**21**	**24**	**28**	**13**	**17**	**21**	**24**	**28**
$\mathrm{Volume}\;\mathrm{Energy}\;\mathrm{Density}\;(ED_{\;vol})$ (mJ·mm^−3^)	208.0	272.0	336.0	400.0	448.0	144.4	188.9	233.3	277.8	311.1	144.4	188.9	233.3	277.8	311.1
Energy Melt Ratio (EMR) (-)	3.3	4.3	5.3	6.3	7.1	2.3	3.0	3.7	4.4	4.9	2.4	3.2	3.9	4.7	5.2

**Table 5:** Optimized theoretical results for $E_{deg_{mod}}$, $EMR_{D_{mod}}$, and $P_{D_{mod}}$ using Equations (8), (9) and (19).

Theoretical Property	First Setting	Second Setting	Third Setting
$\mathrm{Energy}\;\mathrm{for}\;\mathrm{Degradation}\;(E_{deg_{mod}})$ (mJ·mm^−3^)	249.3	249.3	245.5
$\mathrm{Energy}\;\mathrm{Melt}\;\mathrm{Ratio}\;\mathrm{for}\;\mathrm{Degradation}\;(E{MR}_{D_{mod}})$ (-)	3.9	4.1	4.1
$\mathrm{Laser}\;\mathrm{Power}\;\mathrm{for}\;\mathrm{Degradation}\;(P_{D_{mod}})$ (W)	15.6	22.4	22.1

## Data Availability

The raw data are available upon reasonable request to the corresponding author.

## Supplementary Materials

The following supporting information can be downloaded at: https://www.mdpi.com/article/10.3390/ma15010385/s1.
