# Supplementary material for: Setting the Optimal Laser Power for Sustainable Powder Bed Fusion Processing of Elastomeric Polyesters: A Combined Experimental and Theoretical Study
Source: Materials (Basel). 2022 Jan 5;15(1):385. doi: 10.3390/ma15010385 (PMC8746146; doi:10.3390/ma15010385)
Supplement: Supplementary file 1 [file materials-15-00385-s001.zip › materials-1477871-supplementary.pdf]

Supporting Information for "Setting the Optimal Laser Power for Sustainable Powder Bed Fusion Processing of Elastomeric Polyesters: A Combined Experimental and Theoretical Study"

Ruben Vande Ryse [1], Mariya Edeleva [2], Ortwijn Van Stichel [1], Dagmar R. D'hooge [2,3], Frederik Pille [4], Rudinei Fiorio [1], Patrick De Baets [5] and Ludwig Cardon [1,*]

[1] Centre for Polymer and Material Technologies (CPMT), Department of Materials, Textiles and Chemical Engineering, Ghent University, Technologiepark 130, 9052 Zwijnaarde, Belgium; Ruben.VandeRyse@ugent.be (R.V.R.); cpmt@ugent.be (O.V.S.); rudinei.fiorio@UGent.be (R.F.)
[2] Laboratory for Chemical Technology (LCT), Department of Materials, Textiles and Chemical Engineering, Ghent University, Technologiepark 125, 9052 Zwijnaarde, Belgium; mariya.edeleva@ugent.be (M.E.); dagmar.dhooge@ugent.be (D.R.D.)
[3] Centre for Textiles Science and Engineering (CTSE), Department of Materials, Textiles and Chemical Engineering, Ghent University, Technologiepark 70A, 9052 Zwijnaarde, Belgium
[4] Department of Surgery and Anaesthesiology, Faculty of Veterinary Medicine, Ghent University, Saliburylaan 133, 9820 Merelbeke, Belgium; frederik.pille@ugent.be
[5] Soete Laboratory, Departement of Electromechanical, Systems and Metal Engineering, Ghent University, Technologiepark 46, 9052 Zwijnaarde, Belgium; Patrick.DeBaets@UGent.be
* Correspondence: ludwig.cardon@ugent.be

Considering the values of the set laser power (P), the hemispherical reflectance of SLS powders (R_p) (Equation (12) in the main text), the transmittance of light through the particles (T_p) (Equation (15) in the main text), the diameter of the powder particle D, and the diameter of the laser beam D_b, one can evaluate the power absorbed by a solid particle ($P_{p_{solid}}$, W) by:

$$P_{p_{solid}} = \frac{P \cdot (1 - R_p) \cdot (1 - T_p) \cdot \pi(0.5 \times D)^2}{\pi(0.5 \times D_b)^2} \quad \text{(S1)}$$

in which the numerator is the power absorbed by a particle with diameter D ($W{\cdot}m^{-2}$), and the denominator is the total area of the laser beam (m^2).

Similarly, the power absorbed by a molten particle ($P_{p_{melt}}$, W) with an initial diameter D is evaluated replacing R_p in the previous equation by the reflectance of the incident light on the material (R) (Equation (11) in the main text):

$$P_{p_{melt}} = \frac{P \times (1 - R) \times (1 - T_p) \times \pi(0.5 \times D)^2}{\pi(0.5 \times D_b)^2} \quad \text{(S2)}$$

Taking into account the energy for melting of the material, $\left[C_{p_{solid}} \times (T_m - T_b) + \Delta H_m\right]$, the volume of a roughly spherical powder particle with diameter D, $\left[\frac{4}{3}\pi \times (0.5 \times D)^3\right]$, and the specific density of the polymer (ρ), the exposition time to the laser required for melting the solid particle ($t_{mp_{solid}}$, s) is:

$$t_{mp_{solid}} = \frac{\left[C_{p_{solid}} \cdot (T_m - T_b) + \Delta H_m\right] \cdot \left\{\rho \cdot \left[\frac{4}{3}\pi \cdot (0.5 \times D)^3\right]\right\}}{P_{p_{solid}}} \quad \text{(S3)}$$

in which the numerator is the energy for melting a particle with diameter D (J) and the denominator is the power absorbed by the solid particle (W).

If the exposition time of the laser (t_{ex}) (Equation (10) in the main text) is longer than $t_{mp_{solid}}$, the exposition time of the molten particle to the laser ($t_{p_{melt}}$, s) is:

$$t_{p_{melt}} = t_{ex} - t_{mp_{solid}} \quad \text{(S4)}$$

From $t_{p_{melt}}$, $P_{p_{melt}}$, and T_m (°C), the maximal temperature reached in the particle (T_{max}, K) becomes:

$$T_{max} = T_m + \frac{P_{p_{melt}} \times t_{p_{melt}}}{\rho \times \frac{4}{3}\pi \times (0.5 \times D)^3 \times C_{p_{melt}}} \quad \text{(S5)}$$

in which the nominator is the energy absorbed by a molten particle (J) during the time $t_{p_{melt}}$ and the denominator is the energy required to increase the temperature of the particle with 1 °C (J·°C^{-1}).

Considering the volume of a powder particle (V_p), the projected area of the laser beam (Ar_b), and the projected area of the powder particle (Ar_p), Equation (S5) can be rearranged as Equation (18) in the main text:

$$T_{max} = T_m + \left[\frac{P \cdot (1 - R) \cdot (1 - T_p) \cdot Ar_p}{Ar_b \cdot \rho \cdot V_p \cdot C_{p_{melt}}}\right] \cdot \left\{t_{ex} - \frac{(V_p \cdot \rho \cdot Ar_b) \cdot \left[C_{p_{solid}} \cdot (T_m - T_b) + \Delta H_m\right]}{P \cdot (1 - R_p) \cdot (1 - T_p) \cdot Ar_p}\right\} \quad \text{(S6)}$$

From the degradation temperature (T_{deg}, °C) one can calculate the laser power for degradation ($P_{D_{ref+abs}}$, W), i.e., the the laser power required to reach T_{deg}:

$$P_{D_{ref+abs}} = \left\{\frac{V_p \cdot \rho \cdot \left[C_{p_{solid}} \cdot (T_m - T_b) + \Delta H_m\right]}{(1 - R_p) \cdot (1 - T_p)} + \frac{V_p \cdot \rho \cdot \left[C_{p_{melt}} \cdot (T_{deg} - T_m)\right]}{(1 - R) \cdot (1 - T_p)}\right\} \cdot \frac{Ar_b}{Ar_p} \cdot \frac{1}{t_{ex}} \quad \text{(S7)}$$

in which, inside the brackets, the left side of the equation is the energy necessary to melt one particle with diameter D considering the reflected and transmitted light (J), and the right side of the equation is the energy necessary to heat the particle up to T_{deg} considering the reflected and transmitted light (J).

Equation (S7) can be rearranged as Equation (17) in the main text:

$$P_{D_{ref+abs}} = \frac{V_p \cdot \rho \cdot Ar_b \cdot \left[C_{p_{solid}} \cdot (T_m - T_b) + \Delta H_m\right]}{t_{ex} \cdot (1 - R_p) \cdot (1 - T_p) \cdot Ar_p} + \frac{V_p \cdot \rho \cdot Ar_b \cdot C_{p_{melt}} \cdot (T_{deg} - T_m)}{t_{ex} \cdot (1 - R) \cdot (1 - T_p) \cdot Ar_p} \quad \text{(S8)}$$
